# High Electrochemical Performance Phosphorus-Oxide Modified Graphene Electrode for Redox Supercapacitors Prepared by One-Step Electrochemical Exfoliation

**DOI:** 10.3390/nano8060417

**Published:** 2018-06-09

**Authors:** Lei Zu, Xing Gao, Huiqin Lian, Xiaomin Cai, Ce Li, Ying Zhong, Yicheng Hao, Yifan Zhang, Zheng Gong, Yang Liu, Xiaodong Wang, Xiuguo Cui

**Affiliations:** 1 School of Material Science and Engineering, Beijing Institute of Petrochemical Technology, Beijing 102617, China; zulei@bipt.edu.cn (L.Z.); gaoxing@bipt.edu.cn (X.G); lianhuiqin@bipt.edu.cn (H.L.); 13522433853@163.com (X.C.); lice0520@163.com (C.L.); zhongying@bipt.edu.cn (Y.Z.); haoyicheng@bipt.edu.cn (Y.H.); zhangyifan@bipt.edu.cn (Y.Z.); gongzheng@bipt.edu.cn (Z.G); yang.liu@bipt.edu.cn (Y.L.); 2 State Key Laboratory of Organic-Inorganic Composites, Beijing University of Chemical Technology, Beijing 100029, China; wangxd@mail.buct.edu.cn

**Keywords:** graphene, electrochemical anodic exfoliation, phosphorus oxide group, supercapacitors, iodide ions

## Abstract

Phosphorus oxide modified graphene was prepared by one-step electrochemical anodic exfoliation method and utilized as electrode in a redox supercapacitor that contained potassium iodide in electrolytes. The whole preparation process was completed in a few minutes and the yield was about 37.2%. The prepared sample has better electrocatalysis activity for I^−^/I^−^_3_ redox reaction than graphite due to the good charge transfer performance between phosphorus oxide and iodide ions. The maximum discharge specific capacitance is 1634.2 F/g when the current density is 3.5 mA/cm^2^ and it can keep at 463 F/g after 500 charging–discharging cycles when the current density increased about three times.

## 1. Introduction

In recent years, graphene and its composite materials have attracted great attention due to their excellent performance in various fields. Many researchers have presented that graphene plays an important role as electrodes, catalysts, and supports in energy-storage fields, such as supercapacitors, ions batteries, fuel cells and so on [1,2,3,4]. To improve the electrochemical performance of graphene, introducing other active groups (–OH, –COOH, –NO*_x_*, and –PO*_x_*) or heteroatoms, including O, P, N and B, into graphene is regarded as an effective way to adjust the electrochemical properties of graphene. The advantages of this strategy are the heteroatoms could tailor the electronic properties and average electronic charge distribution of adjacent carbon atoms of graphene. Among various heteroatom-doped graphene materials, nitrogen-doped (N-doped) graphene has been widely studied in supercapacitors [5] and batteries [6]. However, only a few studies are reported so far regarding graphene modified with phosphorus (P) and phosphorus-oxide (–PO*_x_*) in electrochemical energy storage field. Recently, Karthika et al. prepared a high electrochemical activity P-doped graphene by reducing graphene oxide with phosphoric acid. The prepared sample exhibited a specific discharge capacitance value of 367 F/g, which is much larger than that of graphene [7]. This study suggests that the electrochemical properties of graphene can be effectively improved by introducing P into graphene. Therefore, it is very necessary to study the preparation and electrochemical properties of P and P–O group modified graphene. In addition, it was revealed that P-modified graphene was much more stable in air than N-modified graphene and exhibited improved n-type semiconducting behavior [8]. However, the traditional preparation methods are relatively complex and time-consuming. Wang et al. synthesized P/C hybrid through a simple ball-milling approach under argon protection. The whole preparation time was 16 h [9]. Xu et al. prepared P doped graphene via thermally annealing approach and the preparation time was more than 48 h [10]. To overcome these drawbacks, it is necessary to use another fast and efficiency method to prepare P modified graphene material. In contrast to the tradition methods, the electrochemical exfoliation approach is believed to be an effective and efficiency strategy. The whole time of preparation usually takes only a few minutes. Accordingly, the preparation of P or P–O group modified graphene through electrochemical approach is more advantageous than traditional methods.

Currently, supercapacitors (SCs) play an important role in the field of energy storage and have been applied in many areas due to high power density, good rate capability and excellent cycling stability [11]. Different from the traditional methods, a unique strategy of introducing redox additives in the electrolytes could effective improve the capacity of SCs via redox reactions at the interface of electrode and electrolyte. Therefore, the efficient use of redox electrolytes is an indispensable way to increase the electrochemical performance of SCs. In our previous studies, we have presented that the potassium iodide (KI) is an effective active redox electrolyte to enhance the capacitance of SCs due to the redox reaction between pairs of 3I^−^/I^−^_3_, 2I^−^/I_2_, 2I^−^_3_/I_2_, I_2_/IO^−^_3_, etc. [12,13,14,15,16,17,18,19]. The electrode materials include conductive polymers such as polyaniline and poly(3,4-ethylenedioxythiophene) (PEDOT):poly(styrene sulfonate) (PSS). Although these polymers have good electrochemical performance, the preparation process is tedious, and, because of the stability restriction of polymer material itself, iodide ions may destroy the structure when they frequently penetrate and release from the polymer. Therefore, it is necessary to apply a new material with good electrochemical performance and that is not easily damaged to improve the performance of the supercapacitor.

In recent studies, some researchers have presented that heteroatom-doped graphene has excellent electrocatalytic activity for the reduction of iodine, due to the electrons conjugation effect, charge redistribution and defect sites [20]. However, the electrochemical catalytic activity of phosphorus oxide modified graphene (PO-graphene) has not been fully revealed. Similar to doping heteroatoms, introducing P–O groups is an effective way to improve the electrochemical performance of graphene. The advantages lie in the modification of the electronic properties and the effect on electronic charge distribution of graphene. Furthermore, the P–O groups could also perform redox reactions with some electroactive ions, such as iodide ions. Therefore, the PO-graphene is a suitable electrode material to use in supercapacitors that contains KI electrolysis.

In this paper, we present a facile method to prepare high quality PO-graphene and study the electrochemical property. The PO-graphene is prepared by the one-step electrochemical anodic exfoliation method and used as the electrode in a redox supercapacitor, whose electrolyte contains KI. We investigated in detail the catalytic effect of P–O group on redox reaction of 3I^−^/I^−^_3_ via various electrochemical analysis. We present that the introduced P–O group in graphene could effectively improve its electrochemical performance. The whole preparation process can be completed within 30 min and the yield is about 37.2%. Owing to the catalytic effect of P–O group, the PO-graphene has a better electrochemical performance than graphite in promoting the redox reaction of I^−^/I^−^_3_.

## 2. Materials and Methods 

### 2.1. Materials

High purity graphite rod was obtained from C Nano Technology Co. Ltd., Beijing, China. Ammonium phosphate, Poly(vinylidene fluoride) (PVDF), Sulfuric acid (H_2_SO_4_) and potassium iodide (KI) were obtained from Aladdin Co. Ltd., Shanghai, China.

### 2.2. Phosphorus Oxide Modified Graphene (PO-Graphene) Preparation

In a typical procedure, high purity graphite rod and platinum plate (1 × 1 × 0.05 cm^3^) were used as the anode and cathode, respectively, and the distance was fixed at 1.5 cm. The exfoliation was performed at 10 V in 1 M ammonium phosphate aqueous. After the preparation, the sample was collected by centrifugation at 5000 rpm for 30 min and purified repeatedly with distilled water until PH = 7.

### 2.3. Characterization

The as-prepared PO-graphene was characterized by field emission scanning electron microscope (FESEM; Zeiss EVO-50, Zeiss, Germany), Transmission electron microscopy (TEM) images are performed on a Tecnal G2 20STWIN microscope operating (FEI, Hillsboro, OR, USA) at 200 kV. Fourier transform Infrared spectroscopy (FT-IR) analyses were accomplished on Thermal Nicolet 6700 (Thermo Nicolet, Shanghai, China). Laser Raman spectra were carried out using Raman spectrometry (RENISHAW inVia Raman Microscope, London, UK). X-ray diffraction analysis (XRD) was carried out by Bruker D8 Advance (BRUKER AXS, Berlin, Germany), Cu Kα = 0.154 nm with a voltage of 40 kV. 

The electrochemical measurements were performed by a three-electrode cell. In detail, the working electrode was composed of PO-graphene (90 wt %) and polyvinylidene fluoride PVDF (10 wt %). A Pt foil electrode and a saturated calomel electrode (SCE) (CH Instruments Ins, Shanghai, China) were used as the counter electrode and reference electrode, respectively. The electrolytes were composed of 0.1 M KI and 0.2 M H_2_SO_4_. The cyclic voltammetry (CV) tests were performed from −0.5 to 0.7 V (vs. SCE). Electrochemical impedance spectroscopy (EIS) measurements were performed under open circuit conditions over a frequency region from 0.01 Hz to 100 kHz by applying an AC signal of 5 mV in amplitude throughout the test. The galvanostatic charge–discharge (GCD) analyses were performed in a potential range of 0–0.7 V (vs. SCE). The CV, GCD and EIS tests were all performed on a CHI660D electrochemical workstation (CH Instruments Ins, Shanghai, China).

## 3. Results

**Figure 1:**
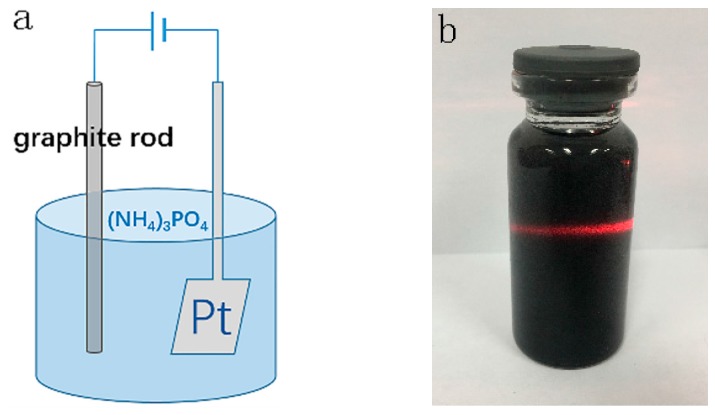

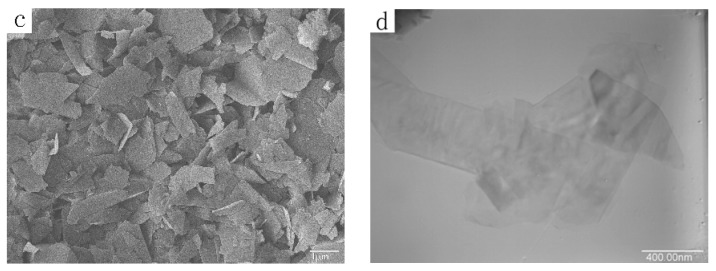
(**a**) schematic diagram of preparation; (**b**) the photo of phosphorus oxide modified graphene (PO-graphene); (**c**) scanning electron microscope (SEM) and (**d**) transmission electron microscopy (TEM) image of PO-graphene.

The schematic diagram of the preparation and the photo of PO-graphene are shown in Figure 1a,b. The preparation process included two parts: oxidation and exfoliation of graphite. The oxidation process produced the P–O groups and then the graphite was exfoliated into few-layer graphene, which possessed a platelet-like morphology (Figure 1c,d), with the promotion of water decomposition. The whole preparation process was completed within 30 min and the yield reached 37.2%.

**Figure 2:**
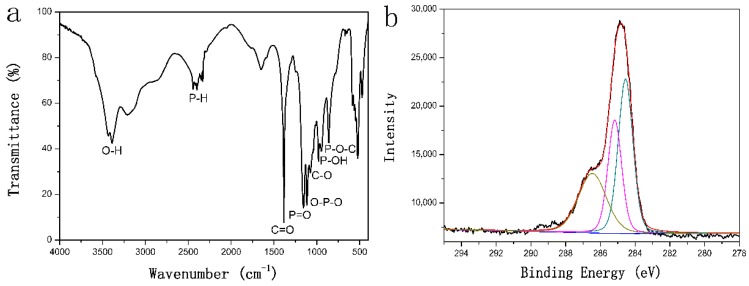

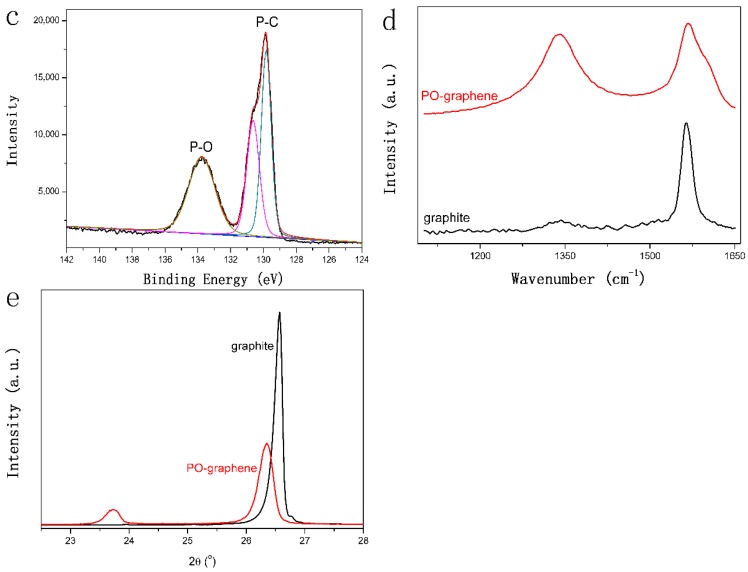
(**a**) Fourier transform Infrared spectroscopy (FT-IR) analysis of PO-graphene; (**b**) high-resolution C1s spectrum of PO-graphene; (**c**) high-resolution P2p spectrum of PO-graphene; (**d**) Raman analysis patterns of PO-graphene and graphite and (**e**) X-ray diffraction analysis (XRD) patterns of PO-graphene and graphite.

The chemical composition of PO-graphene was determined by FT-IR, XPS and Raman spectra. As presented in Figure 2a, the PO-graphene shows clear stretching peaks of P=O, O–P–O, P–OH, and P-H at 1155, 1116, 946, and 2443–2330 cm^−1^, respectively. Different types of oxygen functionalities are presented at 3434 cm^−1^ (O–H stretching), 1076 cm^−1^ (C–O stretching), 980 and 861 cm^−1^ (P–O–C stretching). The peaks at 1649 (C=C skeletal vibrations from graphite) and 2856 cm^−1^ (CH_2_ stretching) are also detected [21]. These characteristic peaks confirm the graphene is successfully modified by P–O group. 

The high resolution XPS spectra could provide detailed bonding and chemical information for the PO-graphene. As presented in Figure 2b, the C 1s peak can be divided into three components centered at about 284.3, 285.8 and 287.5 eV, which cloud be attributed to C–C, C–P, and C–O bonding, respectively [22]. The presence of the C–P characteristic peak confirms that P atoms have been successfully bonded into the graphene. The high-resolution P 2p spectrum (Figure 2c) shows that phosphorus bonded with graphene in two types of chemical bonding: P–C and P–O bonding, located at about 129.8 and 133.7 eV, respectively [23]. The presence of both P–C and P–O stretch models confirms that the P–O group was successfully bonded with graphene.

The Raman spectra of PO-graphene and graphite are presented in Figure 2d. The spectrum of the original graphite shows a very weak D band at 1338 cm^−1^ and strong G band at 1563 cm^−1^. The D band is related to the defects and disorder in the structure of the graphite due to the intervalley scattering, while the G band corresponds to the E_2g_ vibration of sp^2^ of C atoms. In contrast to the origin graphite, the PO-graphene shows a clearly strong D band. The intensity ratio of D to G band (*I*_D_/*I*_G_) increases from 0.1 to 0.87, manifesting that the disorder degree and structural defect (decrease in the average size of the sp^2^ domains) in PO-graphene are enhanced due to the oxidization during the preparation [24]. The decreased layers of graphene in PO-graphene were also demonstrated by the wave shift of the G band from 1563 to 1568 cm^−1^.

The XRD patterns of PO-graphene and graphite are presented in Figure 2e. PO-graphene exhibits two diffraction peaks centered at 23.7° and 26.3°, corresponding to the larger interlayer spacing of 0.375 and 0.338 nm, respectively. Compared with the graphite (about 0.335 nm), the larger interlayer distance is due to the accommodation of oxygen functional groups and water molecules during the preparation due to the obvious hydrophilic character [25]. This result also suggests the formation of P–O groups on graphene.

**Figure 3:**
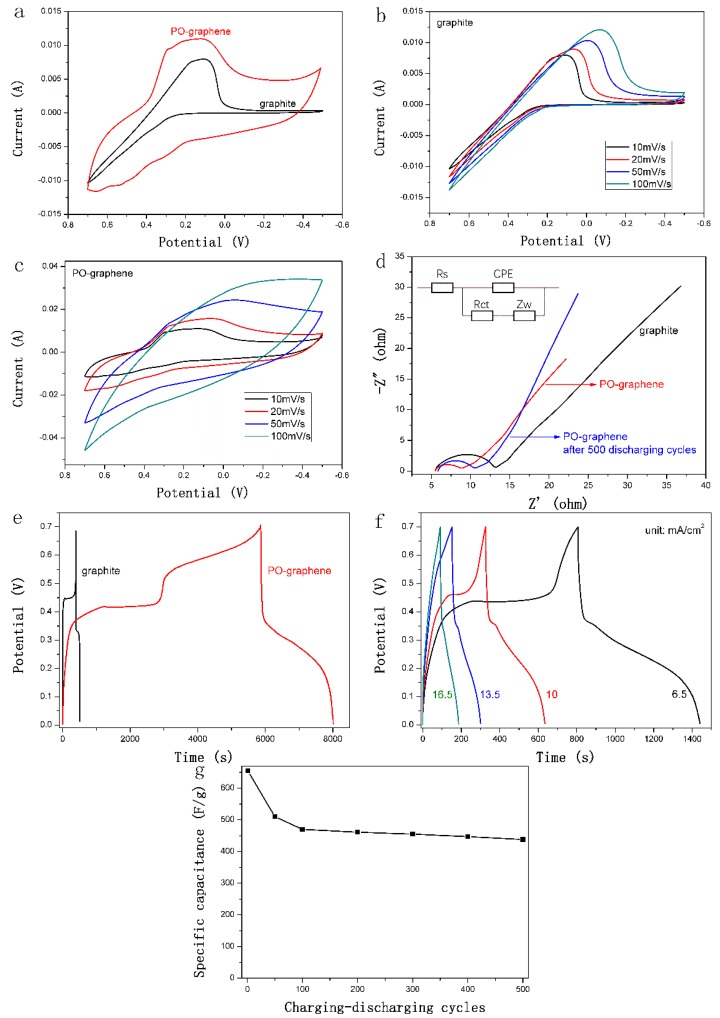
(**a**) Cyclic voltammetry (CV) profiles of PO-graphene and graphite performed in a mixture of 0.1 M KI and 0.2 M H_2_SO_4_ aqueous at 10 mV/s; (**b**) CV profiles of PO-graphene and (**c**) graphite at different scan rate; (**d**) Nyquist plots and equivalent circuit (inset) of PO-graphene and graphite; (**e**) galvanostatic charge–discharge (GCD) analysis of PO-graphene and graphite at 3.5 mA/cm^2^; and (**f**) GCD analysis of PO-graphene at different current density; (**g**) cycling stability analysis at 10 mA/cm^2^.

The electrochemical performance of PO-graphene is investigated by CV analysis. As shown in Figure 3a, compared with the graphite, which possesses only one single reduction peak, the PO-graphene has a more symmetry and larger peak current than that of graphite, suggesting that it has a better electrochemical performance due to the catalysis of P–O groups. The pseudo capacitance performance comes from both the redox transformation of I^−^↔ I^−^_3_ and the interaction between P–O groups and iodide ions. Clearly, the PO-graphene possesses three pair of redox peaks. The peaks centered at 0.27 and 0.12 V are due to the transformation of 3I^−^–2e ↔ I^−^_3_, the peaks at 0.53 and 0.22 V could be assigned to the transformation of 5I^−^–4e ↔ I^−^_5_ and the peaks at 0.65 and 0.29 V may be attributed to the interaction between P–O and iodide ions.

To investigate the charge transfer process between PO-graphene and iodide ions, CV tests at different scan rates were performed. As exhibited in Figure 3b, with increasing scan rate, the separation of the peak potentials was enlarged slightly, indicating that the oxidation and reduction of iodide ions in graphite electrode were surface-controlled process. In contrast, the redox peak current of PO-graphene (Figure 3c) was increased apparently by improving the scan rate and the shape of the curve gradually turns into a rectangle, suggesting that the interaction between PO-graphene and iodide ions was performed through a Faradic reaction. 

The charge transfer performance is also investigated by EIS analysis. The Nyquist plots and equivalent circuit are shown in Figure 3d. In the Nyquist plots, the diameter of the semicircle manifests the difficulty of the charge transfer in the electrochemical system [26]. As can be seen clearly, the PO-graphene shows a smaller semicircle than that of the graphite, suggesting that it has a lower charge transfer resistance and a faster charge transfer process. The charge-transfer resistance for PO-graphene and graphite is 2.09 and 6.67 Ω/cm^2^, respectively. This result could be ascribed to the strong redox interaction between P–O groups and iodide ions, and this conclusion agrees well with the CV results. 

Because of the convenient charge transfer character, the PO-graphene has a better charging–discharging performance than that of graphite. As shown in Figure 3e, the discharge specific capacitance of PO-graphene and graphite is calculated as 1634.2 and 87.1 F/g, respectively. The rate capacity of PO-graphene was tested with different current density. As presented in Figure 3f, the discharge specific capacitance decreased gradually with increasing current density from 6.5 mA/cm^2^ to 16.5 mA/cm^2^ due to the polarization. The maximum discharge specific capacitance was calculated as 904.3 F/g when the current density was 6.5 mA/cm^2^ and it reached 342.5 F/g at 16.5 mA/cm^2^. This result manifests that the PO-graphene possesses a good rate performance and this discharge performance is also better than the other electrochemical systems, as shown in Table 1. The cycling stability of PO-graphene was investigated at the current density of 10 mA/cm^2^. As shown in Figure 3g, the discharge specific capacitance still keeps 438 F/g after 500 charge–discharge cycles. This good cycling stability is ascribed to the excellent charge transfer ability of PO-graphene. The charge-transfer resistance only increased 3.1 Ω/cm^2^ compared to pristine during the charge–discharge process (as presented in Figure 3d, inset).

## 4. Conclusions

In summary, we applied electrochemical anodic exfoliation method to prepare phosphorus oxide modified graphene and used it as an effective electrode in a redox supercapacitor. The PO-graphene has excellent electrochemical performance due to the significant catalysis to iodide ions and convenient charge transfer capability between phosphorus oxide and iodide ions. The maximum discharge capacitance is 1634.2 F/g when the current density is 3.5 mA/cm^2^. The PO-graphene possesses good rate performance and cycling stability.

## Figures and Tables

**Table 1:** Comparison of discharge specific capacitance between the phosphorus oxide modified graphene (PO-graphene) and other reported values.

Materials	Electrolyte	Test Condition	Specific Capacitance (F/g)	Reference
PX-MWCNT	H_2_SO_4_, 1 M	20 mA/cm^2^	118	[27]
PANI/SWCNT	H_2_SO_4_, 1 M	5 mA/cm^2^	485	[28]
PANI/MWCNT	NaNO_3_, 1 M	5 mA/cm^2^	328	[29]
PANI/MWCNT	H_2_SO_4_, 0.5 M	5 mA/cm^2^	500	[15]
N-rGO	H_2_SO_4_, 1 M	1 mA/cm^2^	459	[30]
PEDOT-GF	H_2_SO_4_, 1 M	2 mA/cm^2^	522	[31]
PO-graphene	KI, 0.1 M + H_2_SO_4_, 0.2 M	3.5 mA/cm^2^	1634.2	Present work
		6.5 mA/cm^2^	904.3	
		10 mA/cm^2^	655.7	
		13.5 mA/cm^2^	437.1	
		16.5 mA/cm^2^	342.5	
